# Sorbicillinoids from Fungi and Their Bioactivities

**DOI:** 10.3390/molecules21060715

**Published:** 2016-06-01

**Authors:** Jiajia Meng, Xiaohan Wang, Dan Xu, Xiaoxiang Fu, Xuping Zhang, Daowan Lai, Ligang Zhou, Guozhen Zhang

**Affiliations:** Key Laboratory of Plant Pathology, Ministry of Agriculture/Department of Plant Pathology, College of Plant Protection, China Agricultural University, Beijing 100193, China; mengjiajiax@163.com (J.M.); wangxiaohan99@126.com (X.W.); cauxudan@163.com (D.X.); xiaoxiaofu@cau.edu.cn (X.F.); zhangxuping5@163.com (X.Z.); dwlai@cau.edu.cn (D.L.)

**Keywords:** sorbicillin, sorbicillinoids, bisorbicillinoids, trisorbicillinoids, vertinoids, fungi, occurrence, biological activities

## Abstract

Sorbicillinoids are important hexaketide metabolites derived from fungi. They have a variety of biological activities including cytotoxic, antioxidant, antiviral and antimicrobial activity. The unique structural features of the sorbicillinoids make them attractive candidates for developing new pharmaceutical and agrochemical agents. About 90 sorbicillinoids have been reported in the past few decades. This mini-review aims to briefly summarize their occurrence, structures, and biological activities.

## 1. Introduction

Sorbicillinoids (also called vertinoids) belong to hexaketide metabolites in which the cyclization has taken place on the carboxylate terminus [1]. They have highly diverse bioactivities and have been isolated from either marine [2,3,4] or terrestrial fungi [5,6,7]. Many of them possess elaborate bicyclic or tricyclic systems that appear to arise from the oxidative dearomatizaton and subsequent dimerization/trimerization of sorbicillin (**5**). The presence of the C1’–C6’ sorbyl sidechain is another structural feature of these compounds. The term “sorbicillinoid” has come to encompass the family as a whole and generally refers to any compound that contains the carbon skeleton of sorbicillin.

Since first reported in 1948 by Cram *et al.*, sorbicillinoids have been extensively studied [8,9]. In 2011, Harned and Volp reviewed the structures of 62 sorbicillinoids [1]. Since then, many new members of this family were isolated and great progress has been made [4,10,11,12,13]. According to the structural features, sorbicillinoids can be divided into four groups: monomeric sorbicillinoids, bisorbicillinoids, trisorbicillinoids, and hybrid sorbicillinoids. Biosynthesis and chemical synthesis have been extensively studied and reviewed [1,11,14,15,16,17]. In this mini-review, we focus on the occurrence and biological activities of sorbicillinoids, and 28 additional sorbicillinoids were added on the basis of the previous review [1].

## 2. Occurrence

**Figure 1:**
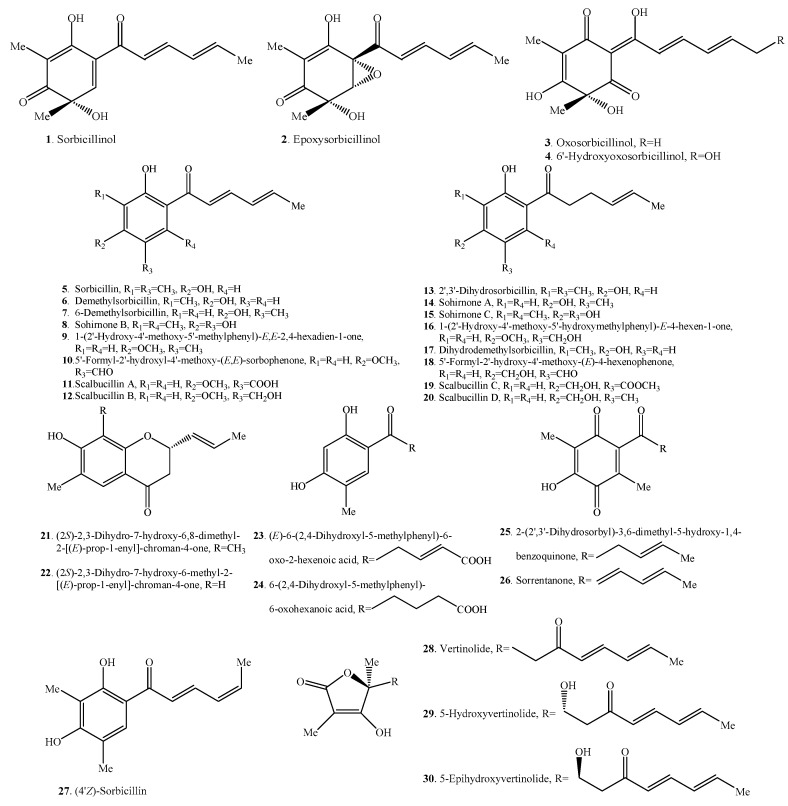
Structures of the monomeric sorbicillinoids (**1**–**30**) isolated from fungi.

**Figure 2:**
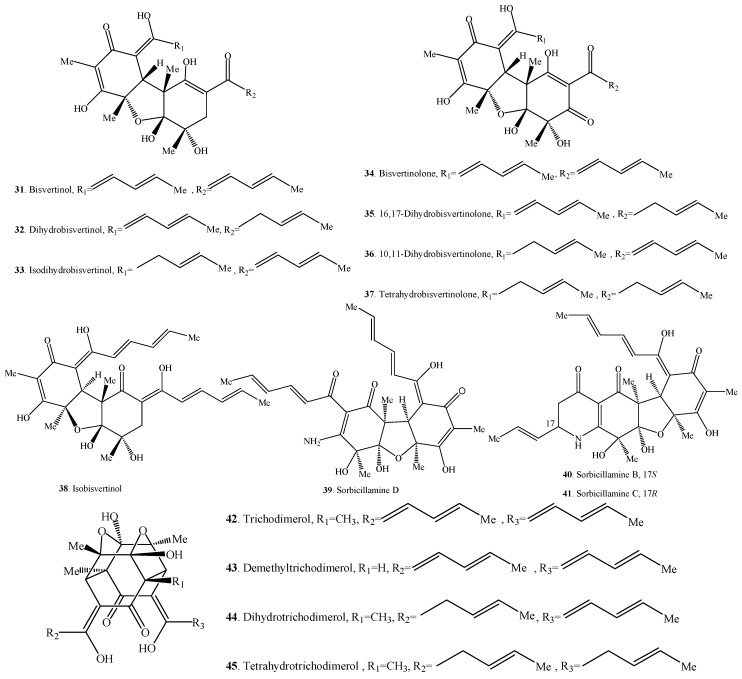

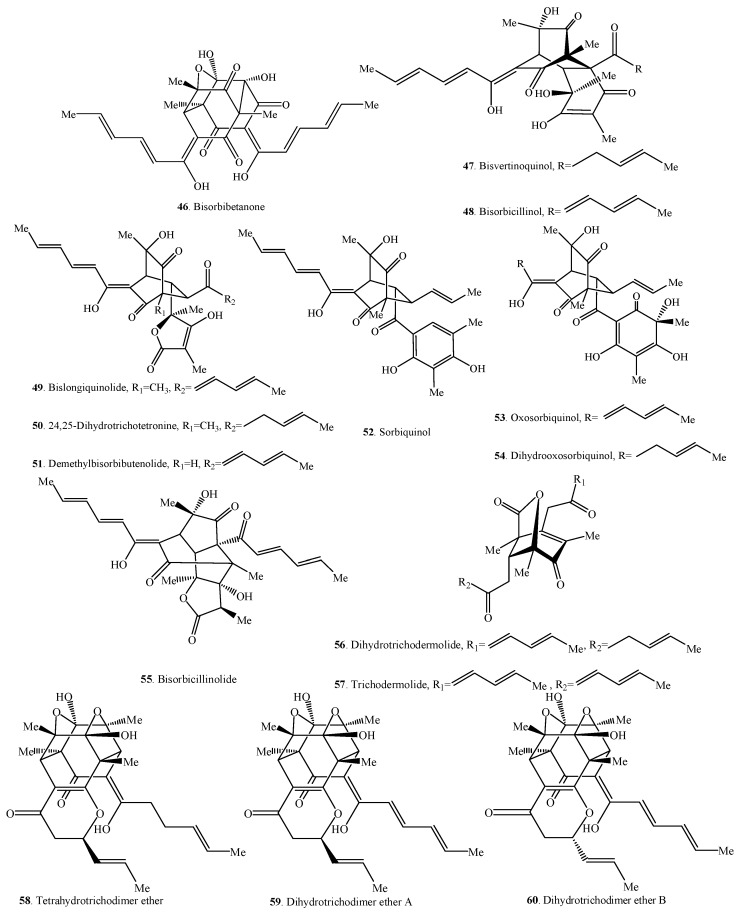
Structures of the bisorbicillinoids (**31**–**60**) isolated from fungi.

**Figure 3:**
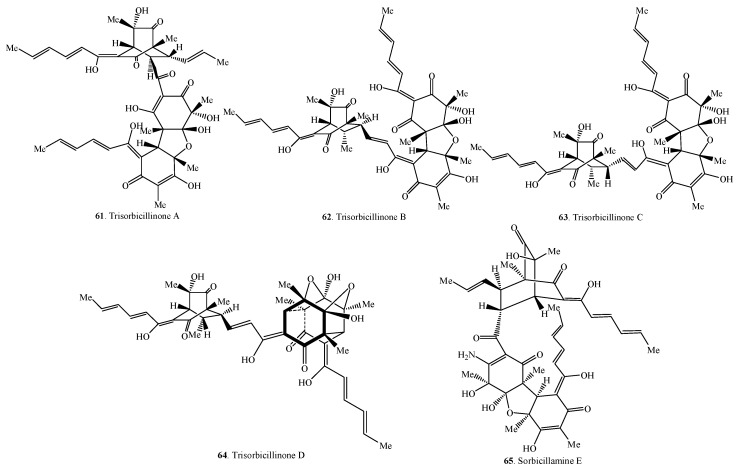
Structures of the trimeric sorbicillinoids (**61**–**65**) isolated from fungi.

**Figure 4:**
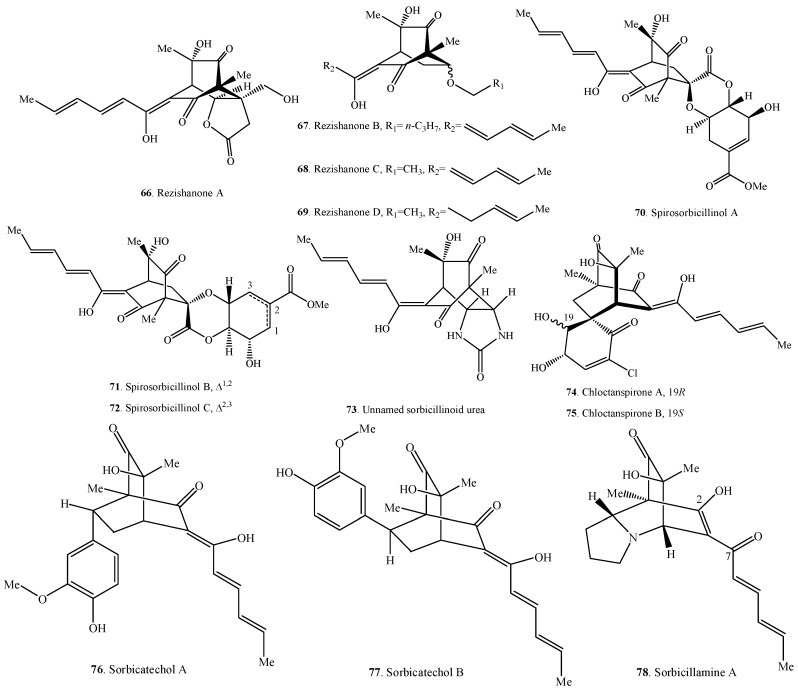

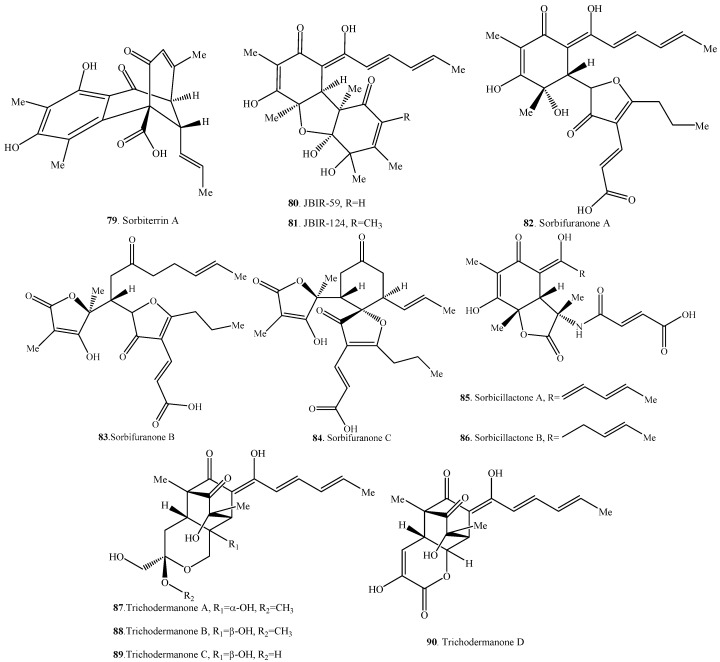
Structures of the hybrid sorbicillinoids (**66**–**90**) isolated from fungi.

Sorbicillinoids have a diverse distribution in fungi (Table 1, Table 2, Table 3 and Table 4). Accordingly, their structures are shown in Figure 1, Figure 2, Figure 3 and Figure 4. In total, about 90 sorbicillinoids have been isolated, and they were found mainly in terrestrial fungi, which contained nine genera, namely *Acremonium*, *Aspergillus*, *Clonostachys, Emericella,* *Penicillium*, *Phaeoacremonium*, *Scytalidium*, *Trichoderma*, and *Verticillium*, and partly in marine fungi that included five genera (*i.e*., *Paecilomyces*, *Penicillium*, *Phialocephala*, *Trichoderma* and *Trichothecium*). All these fungi belong to ascomycetes.

### 2.1. Monomeric Sorbicillinoids

To date, 30 monomeric sorbicillinoids (Table 1 and Figure 1) have been isolated from *Clonostachys*, *Emericella*, *Penicillium*, *Phaeoacremonium*, *Phialocephala*, *Scytalidium*, *Trichoderma*, *Trichothecium* and *Verticillium* species.

Sorbicillinol (**1**) was found to be highly reactive and it was the biosynthetic precursor of the other sorbicillinoid family members [11].

Sorrentanone (=3-hydroxy-2,5-dimethyl-6-(1′-oxo-2′,4′-dienylhexyl)-1,4-benzoquinone, **26**) was the benzoquinone structure of sohirnone B (**8**), meaning that it was imagined arising from the oxidation of sohirnone B (**8**) [5,18]. Similarly, 2-(2′,3′-dihydrosorbyl)-3,6-dimethyl-5-hydroxy-1,4-benzoquinone (**25**) was the benzoquinone of sohirnone C (**15**) [5,19].

### 2.2. Bisorbicillinoids

Bisorbicillinoids are also called dimeric sorbicillinoids, which consist of two sorbicillinoid monomers (Table 2), whose structures are shown in Figure 2. Up to now, 30 bisorbicillinoids have been isolated from fungi. These compounds are mainly distributed in the genera *Acremonium*, *Aspergillus*, *Clonostachys*, *Penicillium*, *Phialocephala*, *Trichoderma*, *Trichothecium* and *Verticillium.*

### 2.3. Trisorbicillinoids

Trisorbicillinoids are also called trimeric sorbicillinoids. Up to date, only five trimeric sorbicillinoids have been isolated from marine fungi (*i.e*., *Penicillium* sp. F23-2 and *Phialocephala* sp. FL30r) (Table 3 and Figure 3). Among them, sorbicillamine E (**65**) was a compound containing N element [10].

### 2.4. Hybrid Sorbicillinoids

Hybrid sorbicillinoids are proposed to be derived from either a Diels-Alder or a Michael reaction of a monomeric sorbicillinoid diene and a second non-sorbicillinoid dienophile. About 25 hybrid sorbicillinoids have been isolated from fungi so far.

The structure of sorbicillamine A (**78**) was a tentative assignment for the C-2/C-7 unit, which might exist as either enol or keto tautomers, and they were interconverting on the NMR timescale in solution [10].

Compound **73** from an intertidal marine fungus *Paecilomyces marquandii* was an unnamed sorbicillinoid urea [57]. Chloctanspirones A (**74**) and B (**75**) containing chlorine were isolated from *Penicillium terrestre* derived from a marine sediment. The differences between them were their absolute configuration at C-19 [58]. Similarly, both sorbicatechols A (**76**) and B (**77**) were isolated from the marine sediment-derived fungus *Penicillium chrysogenum* PJX-17, and their differences were the absolute configuration at C-7 [59].

Unnamed urea (**73**), sorbicillamine A (**78**), sorbicillactone A (**85**), and sorbicillactone B (**86**) were a class of N-containing compounds [10,21,57]. Interestingly, the N-containing sorbicillinoids including dimeric sorbicillamines D (**39**), B (**40**), C (**41**), and trimeric sorbicillamine E (**65**) were all isolated from marine fungi (Table 2, Table 3 and Table 4). Except urea **73** from the genus *Paecilomyces*, others were isolated from the genus *Penicillium*.

## 3. Biological Activities

### 3.1. Cytotoxic Activity

Many sorbicillinoids were screened to have cytotoxic activities, which are summarized in Table 5. (2*S*)-2,3-Dihydro-7-hydroxy-6,8-dimethyl-2-[(*E*)-prop-1-enyl]-chroman-4-one (**21**) and (2*S*)-2,3-dihydro-7-hydroxy-6-methyl-2-[(*E*)-prop-1-enyl]-chroman-4-one (**22**) displayed significant activities against the human breast cancer cell line MCF-7 with IC_50_ values of 9.51 and 7.82 μg/mL, respectively, and 2′,3′-dihydrosorbicillin (**13**) showed moderate cytotoxicity against various human cancer cell lines (colon cancer cell line Lovo, hepatic cancer cell line Bel-7402, lung cancer line A549, nasopharyngeal carcinoma cell lines CNE1, CNE2, KB and SUNE1) with IC_50_ values ranging from 9.19 to 21.93 μg/mL [4].

5′-Formyl-2′-hydroxyl-4′-methoxy-(*E*,*E*)-sorbophenone (**10**) showed cytotoxic activity on OSU-CLL (lymphocytic leukemia) cell lines with IC_50_ value of 3.1 µM at 48 h, on MDA-MB-435 (melanoma) and SW-620 (colon) cell lines with IC_50_ values of 1.5 and 0.5 µM at 72 h, respectively. Similarly, 1-(2′-hydroxy-4′-methoxy-5′-methylphenyl)-*E*,*E*-2,4-hexadien-1-one (**9**) on MDA-MB-435 and SW-620 cell lines with IC_50_ values of 65.2 and 15.1 µM, scalbucillin B (**12**) on MDA-MB-435 and SW-620 cell lines with IC_50_ values of 67.9 and 16.0 µM, and 5′-formyl-2′-hydroxy-4′-methoxy-(*E*)-4-hexenophenone (**18**) on MDA-MB-435 and SW-620 cell lines with IC_50_ values of 2.3 and 2.5 µM at 72 h, respectively [12].

(*E*)-6-(2,4-Dihydroxyl-5-methylphenyl)-6-oxo-2-hexenoic acid (**23**) and 6-(2,4-dihydroxyl-5-methylphenyl)-6-oxohexanoic acid (**24**) from a saline lands-derived fungus *Trichoderma* sp. showed cytotoxic effects on P388 cell line with IC_50_ values of 72.8 and 44.5 μM, and on HL-60 cell line with IC_50_ values of 52.5 and 81.2 μM, respectively [6].

Dihydrotrichodermolide (**56**) and dihydrodemethylsorbicillin (**17**) displayed cytotoxic effects against P388 cell line (IC_50_ values of 11.5 and 0.1 μM, respectively) and K562 cell line (IC_50_ values of of 22.9 and 4.8 μM, respectively) [36].

Chloctansprirone A (**74**) was active against HL-60 and A549 cell lines with IC_50_ values of 9.2 and 39.7 μM, respectively. Chloctansprirone B (**75**) showed relatively weak activity against HL-60 cells with IC_50_ value of 37.8 μM [58].

By comparing the structure-activity relationships of the compounds, the sorbyl sidechain was very important. Sorbicillinoids with their C_2_′-C_3_′ double bond being reduced were less active. For example, sorbicllin (**5**) showed significant inhibitory activity on HeLa and HepG2 cells with IC_50_ values of 1.6 and 27.2 μM, respectively. On the contrary, 2′,3′-dihydrosorbicillin (**13**) with the C_2_′-C_3_′ double bond being reduced showed less activity on HeLa and HepG2 cells with IC_50_ values of 7.4 and 44.4 μM, respectively. The same phenomena were observed for the compounds 6-demethylsorbicillin (**7**) *vs.* sohirnone A (**14**) [27], bisvertinolone (**34**) *vs.* 10,11-dihydrobisvertinolone (**36**) [27], and 5′-formyl-2′-hydroxyl-4′-methoxy-(*E,E*)-sorbophenone (**10**) *vs.* 5′-formyl-2′-hydroxy-4′-methoxy-(*E*)-4-hexenophenone (**18**) [12].

### 3.2. Antimicrobial Activity

Some sorbicillinoids exhibited antimicrobial activities that are shown in Table 6. 5′-Formyl-2′-hydroxyl-4′-methoxy-(*E*,*E*)-sorbophenone (**10**) and 5′-formyl-2′-hydroxy-4′-methoxy-(*E*)-4-hexenophenone (**18**) displayed strong antifungal activity on *A. niger* with MIC values of 0.05 and 0.04 μg/mL (0.20 and 0.16 μM), respectively, much more potent than the positive control (amphotericin B, MIC value of 31 μg/mL). Scalbucillin B (**12**) showed an MIC value of 0.60 μg/mL (2.42 μM) against *Aspergillus niger*. Considering the potent antimicrobial activity, a hemolytic assay toward sheep red blood cells *in vitro* was carried out to assess the toxicity of these compounds (**10**, **12**, **18**). They showed a similarly low toxicity on sheep red blood cells, which indicated the promising safety for their potential application as the anti-*Aspergillus* agents [12].

Dihydrotrichodimerol (**44**) and tetrahydrotrichodimerol (**45**) exhibited strong antibacterial activity on *Bacillus megaterium* with MIC values of 25 and 12.5 μg/mL, respectively. Dihydrotrichodimer ether A (**59**) and dihydrotrichodimer ether B (**60**) had strong antibacterial activity on *Escherichia coli* with MIC values of 25 and 50 μg/mL, respectively. Furthermore, dihydrotrichodimer ether B (**60**) showed preferable antibacterial activity against *Ballus subtilis* with MIC value of 50 μg/mL [13].

### 3.3. Antiviral Activity

Sorbicatechols A (**76**) and B (**77**) from the marine-derived fungus *Penicillium chrysogenum* PJX-17 showed potent antiviral activity against influenza A virus (H1N1) with IC_50_ values of 85 and 113 μM, respectively (ribavirin as the positive control with IC_50_ value of 84 μM) [59].

Sorbicillactone A (**85**) from a sponge-derived fungus *Penicillium chrysogenum* displayed anti-HIV activity. It protected human T lymphocytes (H9 cells) against the cytopathic effect of HIV-1 in the concentration range of 0.3 and 3.0 μg/mL [21]. This hybrid sorbicillinoid was considered to be a potential inhibitor to VP40 matrix protein of the Ebola virus [63].

### 3.4. Antioxidant Activity

Active oxygen species cause many diseases such as atherosclerosis, inflammation, ischemia-reperfusion injury, rheumatioid arthritis and central nervous diseases. Furthermore, senility, cancer initiation and progression are also believed to involve active oxygen species [64,65]. Thus, it is expected that the effective antioxidant agents may prevent the onset and development of these diseases. Some sorbicillinoids exhibited obviously antioxidant activity. The DPPH radical scavenging activity of the sorbicillinoids isolated before 2011 was well summarized [1]. After 2011, only one sorbicillinoid JBIR-124 (**81**) from *Penicillium citrinum* Sp1080624G1f01 was screened to have DPPH radical scavenging activity with IC_50_ value of 30 µM [62].

### 3.5. Other Biological Activities

Other biological activities of the sorbicillinoids are shown in Table 7. Dihydrotrichodimerol (**44**) and bislongiquinolide (=bisorbibutenolide=trichotetronine, **49**) from *Trichoderma citrinoviridev* influenced aphid feeding preferences [48]. Isobisvertinol (**38**) from *Aspergillus* sp. FKI-1746 inhibited lipid droplet accumulation in macrophages [40].

In addition, dihydrotrichodimerol (**44**) from an unidentified fungus activated peroxisome proliferator-activated receptor γ (PPAR γ) with an ED_50_ value of 80 ng/mL [50]. Bisvertinolone (**34**) from *Verticillium intertextum* inhibited the biosynthesis of β-l,6-glucan [42].

Trichodimerol (=MS-182123, **42**) from *Penicillium chrysogenum* strain V39673 inhibited the production of tumor necrosis factor-α (TNF-α) by macrophages (IC_50_ value of 200 ng/mL) and monocytes (IC_50_ value of 200 ng/mL) [46]. Subsequently, trichodimerol was screened to show an inhibitory effect on lipopolysaccharide-induced eicosanoid secretion in THP-1 human monocytic cells [66].

6′-Hydroxyoxosorbicillinol (**4**) showed inhibition on soybean lipoxygenase activity with an IC_50_ value of 16 µM, about 10 folds higher than oxosorbicillinol (**3**). 6′-Hydroxyoxosorbicillinol (**4**) also exhibited prostaglandin D_2_ and leukotriene B_4_ release suppression activity with IC_50_ values of 10 and 100 µM, respectively [22].

Sorbiterrin A (**79**) showed moderate acetylcholinesterase (AChE) inhibitory effect with IC_50_ value of 25 µg/mL [61].

## 4. Conclusions

About 90 sorbicillinoids have been isolated from terrestrial and marine ascomycetous fungi in the past few decades. Some of them exhibited promising bioactivities, especially cytotoxic, antioxidant, antimicrobial, and antiviral activities. In recent years, more and more new members of sorbicillinoids have been isolated. All these sorbicillinoids could be the rich resources of biologically active substances with significant medicinal and agricultural potential.

The biosynthesis studies of sorbicillinoids have been carried out [11,14,15,16,17] and well summarized [1]. Sorbicillinol (**1**) has been hypothesized as a precursor of most sorbicillinoids that were biosynthesized by polyketide synthases (PKs) [14]. In addition, the PKS gene cluster containing *SorbA, SorbB* and *SorbC* has been characterized for sorbicillin (**5**) biosynthesis, and sorbicillinol (**1**) was proved as a key intermediate [11]. The extensive ^13^C enrichment studies carried out by Abe and co-workers have unequivocally demonstrated that many of biosynthetic hypotheses of sorbicillinoids are correct [14,15,16,17]. There are still some uncertainties. Furthermore, the specific polyketide synthases in the biosynthetic pathway of sorbicillinoids in fungi have not been characterized. Chemical syntheses of sorbicillinoids have attracted pharmaceutical chemists as they have potential applications in the agriculture, pharmaceutical and food industries. Some sorbicillinoids such as sorbicillin (**5**), vertinolide (**28**), epoxysorbicillinol (**2**), and trichodimerol (=MS-182123, **42**) have been synthesized successfully, and well summarized [1].

In most cases, biological activities, structure-activity relations, and mode of action of sorbicillinoids have been investigated based on *in vitro* studies or animal models. Few studies have been performed at the level of clinical trials in patients. Future studies should be emphasized on the improvement in methodological quality and warrant further clinical research on the effects of these compounds. The applications of sorbicillinoids as antitumor agents, antimicrobials, antivirus agents and antioxidants, as well as their underlying bioactivities, have led to considerable interest within the pharmaceutical community and health-care industry. With a good understanding of the biosynthetic pathways of some sorbicillinoids, we can not only increase outputs of the bioactive sorbicillinoids but also block biosynthesis of some harmful sorbicillinoids by specific interferences.

## Figures and Tables

**Table 1:** Occurrence of the monomeric sorbicillinoids (**1**–**30**) in fungi.

Sorbicillinoid	Fungus and its Origin	Ref.
Sorbicillinol (**1**)	*Trichoderma* sp. USF-2690 from a soil sample	[14]
Epoxysorbicillinol (**2**)	*Trichoderma longibrachiatum* from the sponge *Haliclona* sp.	[20]
Oxosorbicillinol (**3**)	*Penicillium chrysogenum* E01-10/3 from the sponge *Ircinia fasciculata*	[21]
	*Penicillium notatum* from a benchtop contamination	[5]
	*Penicillium* sp. 06T121 from a soil sample	[22]
	*Trichoderma* sp. USF-2690 from a soil sample	[23]
6′-Hydroxyoxosorbicillinol (**4**)	*Penicillium* sp. 06T121 from a soil sample	[22]
Sorbicillin (**5**)	*Clonostachys rosea* YRS-06 from a soil sample	[13]
	*Emericella* sp. IFM57991 and its origin was not clear	[24]
	*Penicillium chrysogenum* Q176 and its origin was not clear	[25]
	*Penicillium chrysogenum* E01-10/3 from the sponge *Ircinia fasciculata*	[11,21]
	*Penicillium notatum* and its origin was not clear	[8,9]
	*Penicillium* sp. P-1 as an endophyte from the stems of *Huperzia serrata*	[7]
	*Trichoderma longibrachiatum* UAMH 4159 and its origin was not clear	[26]
	*Trichoderma* sp. from the seastar *Acanthaster planci*	[4]
	*Trichoderma* sp. f-13 from a marine sediment	[27]
	*Trichoderma* sp. PR-35 as an endophyte from *Paeonia delavayi*	[28]
	*Trichoderma* sp. USF-2690 from a soil sample	[29]
	*Trichothecium* sp*.* from a marine sediment	[30]
	*Verticillium intertextum* and its origin was not clear	[31,32]
Demethylsorbicillin (**6**)	*Trichoderma* sp. USF-2690 from a soil sample	[23]
6-Demethylsorbicillin (**7**)	*Trichoderma* sp. f-13 from a marine sediment	[27]
Sohirnone B (**8**)	*Penicillium notatum* from a benchtop contamination	[5]
1-(2′-Hydroxy-4′-methoxy-5′-methylphenyl)-*E,E*-2,4-hexadien-1-one (**9**)	*Phaeoacremonium* sp. NRRL32148 from the surface of stromata of *Hypoxylon truncatum* formed on a dead hardwood branch	[33]
	*Scytalidium album* MSX51631 from a soil sample	[12]
5′-Formyl-2′-hydroxyl-4′-methoxy-(*E,E*)-sorbophenone (**10**)	*Phaeoacremonium* sp. NRRL32148 from the surface of stromata of *Hypoxylon truncatum* formed on a dead hardwood branch	[33]
	*Scytalidium album* MSX51631 from a soil sample	[12]
	*Scytalidium* sp. FY as an immunizing commensal of Douglasfir utility poles	[34]
Scalbucillin A (**11**)	*Scytalidium album* MSX51631 from a soil sample	[12]
Scalbucillin B (**12**)	*Scytalidium album* MSX51631 from a soil sample	[12]
2′,3′-Dihydrosorbicillin (**13**)	*Penicillium chrysogenum* R03-8/4 from the sponge *Tethya aurantium*	[35]
	*Penicillium chrysogenum* E01-10/3 from the sponge *Ircinia fasciculata*	[11]
	*Penicillium notatum* from a benchtop contamination	[5]
	*Penicillium* sp. P-1 as an endophyte from the stems of *Huperzia serrata*	[7]
	*Trichoderma* sp. from the seastar *Acanthaster planci*	[4]
	*Trichoderma* sp. f-13 from a marine sediment	[27]
	*Verticillium intertextum* from a laboratory contaminant	[31,32]
Sohirnone A (**14**)	*Penicillium notatum* from a benchtop contamination	[5]
	*Trichoderma* sp. f-13 from a marine sediment	[27]
Sohirnone C (**15**)	*Penicillium notatum* from a benchtop contamination	[5]
1-(2′-Hydroxy-4′-methoxy-5′-hydroxymethylphenyl)-*E*-4-hexen-1-one (**16**)	*Phaeoacremonium* sp. from the surface of stromata of *Hypoxylon truncatum* formed on a dead hardwood branch	[33]
	*Scytalidium album* MSX51631 from a soil sample	[12]
Dihydrodemethylsorbicillin (**17**)	*Phialocephala* sp. FL30r from a deep sea sediment	[36]
5′-Formyl-2′-hydroxy-4′-methoxy-(*E*)-4-hexenophenone (**18**)	*Scytalidium album* MSX51631 from a soil sample	[12]
	*Scytalidium* sp. FY as an immunizing commensal of Douglasfir utility poles	[34]
Scalbucillin C (**19**)	*Scytalidium album* MSX51631 from a soil sample	[12]
Scalbucillin D (**20**)	*Scytalidium album* MSX51631 from a soil sample	[12]
(2*S*)-2,3-Dihydro-7-hydroxy-6,8-dimethyl-2-[(*E*)-prop-1-enyl]-chroman-4-one (**21**)	*Trichoderma* sp. from the seastar *Acanthaster planci*	[4]
	*Penicillium* sp. P-1 as an endophyte from the stems of *Huperzia serrata*	[7]
(2*S*)-2,3-Dihydro-7-hydroxy-6-methyl-2- [(*E*)-prop-1-enyl]-chroman-4-one (**22**)	*Trichoderma* sp. from the seastar *Acanthaster planci*	[4]
(*E*)-6-(2,4-Dihydroxyl-5-methylphenyl)-6-oxo-2-hexenoic acid (**23**)	*Trichoderma* sp. JH8 from the soil of saline lands	[6]
6-(2,4-Dihydroxyl-5-methylphenyl)-6-oxohexanoic acid (**24**)	*Trichoderma* sp. JH8 from the soil of saline lands	[6]
2-(2′,3′ -Dihydrosorbyl)-3,6-dimethyl-5-hydroxy-1,4-benzoquinone (**25**)	*Penicillium terrestre* from a marine sediment	[19]
Sorrentanone = 3-hydroxy-2,5-dimethyl-6-(1′-oxo-2′,4′-dienylhexyl)-1,4-benzoquione (**26**)	*Penicillium chrysogenum* SC13887 and its origin was not clear	[18]
(4’*Z*)-Sorbicillin (**27**)	*Trichoderma* sp. from the seastar *Acanthaster planci*	[4]
Vertinolide (**28**)	*Trichoderma viride* from the sponge *Agelas dispar*	[3]
	*Trichoderma* sp. from the sponge *Agelas dispar*	[37]
	*Verticillium intertextum* from a laboratory contaminant	[31,38]
5-Hydroxyvertinolide (**29**)	*Trichoderma longibrachiatum* UAMH 4159 and its origin was not clear	[39]
5-Epihydroxyvertinolide (**30**)	*Trichoderma* sp. USF-2690 from a soil sample	[17]

Note: Compounds **4**, **11**, **12** and **17**–**24** were not included in the last review [1].

**Table 2:** Occurrence of the bisorbicillinoids (**31**–**60**) in fungi.

Sorbicillinoid	Fungus and Its Origin	Ref.
Bisvertinol (**31**)	*Aspergillus* sp. FKI-1746 from a mangrove slurry sample	[40]
	*Trichoderma longibrachiatum* UAMH 4159 and its origin was not clear	[26]
	*Trichoderma viride* from the sponge *Agelas dispar*	[3]
	*Trichoderma* sp. from the sponge *Agelas dispar*	[37]
	*Verticillium intertextum* from a laboratory contaminant	[41]
Dihydrobisvertinol (**32**)	*Aspergillus* sp. FKI-1746 from a mangrove slurry sample	[40]
	*Verticillium intertextum* from a laboratory contaminant	[41]
Isodihydrobisvertinol (**33**) Bisvertinolone (**34**)	*Verticillium intertextum* from a laboratory contaminant	[41]
	*Acremonium strictum* and its origin was not clear	[42]
	*Penicillium chrysogenum* E01-10/3 from the sponge *Ircinia fasciculata*	[21]
	*Penicillium citrinum* SpI080624G1f01 from a marine sponge	[43]
	*Penicillium notatum* from a benchtop contamination	[5]
	*Trichoderma longibrachiatum* UAMH 4159 and its origin was not clear	[26]
	*Trichoderma* sp. f-13 from a marine sediment	[27]
	*Trichoderma* sp. JH8 from the soil of saline lands	[6]
	*Trichoderma* sp. USF-2690 isolated from a soil sample	[44]
	*Verticillium intertextum* from a laboratory contaminant	[41]
16,17-Dihydrobisvertinolone (**35**)	*Penicillium terrestre* from a marine sediment	[19]
10,11-Dihydrobisvertinolone (**36**)	*Trichoderma* sp. f-13 from a marine sediment	[27]
Tetrahydrobisvertinolone (**37**)	*Penicillium terrestre* from a marine sediment	[19]
Isobisvertinol (**38**)	*Aspergillus* sp. FKI-1746 from a mangrove slurry sample	[40]
Sorbicillamine D (**39**)	*Penicillium* sp. F23-2 from a deep-sea sediment	[10]
Sorbicillamine B (**40**)	*Penicillium* sp. F23-2 from a deep-sea sediment	[10]
Sorbicillamine C (**41**)	*Penicillium* sp. F23-2 from a deep-sea sediment	[10]
Trichodimerol = MS-182123 (**42**)	*Clonostachys rosea* YRS-06 from a soil sample	[13]
	*Penicillium chrysogenum* V39673 and its origin was not clear	[45,46]
	*Penicillium citrinum* SpI080624G1f01 from a marine sponge	[43]
	*Penicillium terrestre* from a marine sediment	[47]
	*Trichoderma citrinoviride* ITEM 4484 from the soil under the tree *Abies* sp.	[48]
	*Trichoderma viride* from the sponge *Agelas dispar*	[3]
	*Trichoderma longibrachiatum* UAMH 4159 and its origin was not clear	[26]
	*Trichoderma* sp. from the straws of rice	[49]
	*Trichoderma* sp. from the sponge *Agelas dispar*	[37]
	*Trichoderma* sp. f-13 from a marine sediment	[27]
	*Trichoderma* sp. JH8 from the soil of saline lands	[6]
	*Trichoderma* sp. USF-2690 from a soil sample	[44]
	*Trichothecium* sp*.* from a marine sediment	[30]
	Unidentified fungus B00853 from a soil sample	[50]
Demethyltrichodimerol (**43**)	*Trichoderma* sp. USF-2690 isolated from a soil sample	[44]
Dihydrotrichodimerol (**44**)	*Clonostachys rosea* YRS-06 from a soil sample	[13]
	*Penicillium terrestre* from a marine sediment	[47]
	*Trichoderma citrinoviride* ITEM 4484 from the soil under the tree *Abies* sp.	[48,51]
	*Trichoderma* sp. f-13 from a marine sediment	[27]
	Unidentified fungus B00853 from a soil sample	[50]
Tetrahydrotrichodimerol (**45**)	*Clonostachys rosea* YRS-06 from a soil sample	[13]
	*Penicillium terrestre* from a marine sediment	[47]
Bisorbibetanone (**46**)	*Trichoderma* sp. USF-2690 isolated from a soil sample	[52]
Bisvertinoquinol (**47**)	*Penicillium notatum* from a benchtop contamination	[5]
	*Trichoderma* sp. f-13 from a marine sediment	[27]
	*Verticillium intertextum* from a laboratory contaminat	[31,32]
Bisorbicillinol (**48**)	*Penicillium notatum* from a benchtop contamination	[5]
	*Trichoderma* sp. f-13 from a marine sediment	[27]
	*Trichoderma* sp. USF-2690 from a soil sample	[44]
Bislongiquinolide = Bisorbibutenolide = Trichotetronine (**49**)	*Penicillium citrinum* SpI080624G1f01 from the sponge *Demospongiae* sp.	[43]
	*Trichoderma citrinoviride* ITEM 4484 from the soil under the tree *Abies* sp.	[48,51]
	*Trichoderma longibrachiatum* from the sponge *Haliclona* sp.	[20]
	*Trichoderma longibrachiatum* UAMH 4159 and its origin was not clear	[26,39]
	*Trichoderma viride* from the sponge *Agelas dispar*	[3]
	*Trichoderma* sp. from the straws of rice plant	[49]
	*Trichoderma* sp. from the sponge *Agelas dispar*	[37]
	*Trichoderma* sp. f-13 from a marine sediment	[27]
	*Trichoderma* sp. USF-2690 from a soil sample	[29]
24,25-Dihydrotrichotetronine = 16,17-Dihydrobislongiquinolide (**50**)	*Trichoderma citrinoviride* ITEM 4484 from the soil under the tree *Abies* sp.	[48,51]
	*Trichoderma* sp. from the straws of rice plant	[49]
Demethylbisorbibutenolide (**51**)	*Trichoderma* sp. USF-4860 from a soil sample	[53]
Sorbiquinol (**52**)	*Trichoderma longibrachiatum* UAMH 4159 and its origin was not clear	[26,54]
Oxosorbiquinol (**53**)	*Phialocephala* sp. FL30r from a deep-sea sediment	[2]
Dihydrooxosorbiquinol (**54**)	*Phialocephala* sp. FL30r from a deep-sea sediment	[2]
Bisorbicillinolide (**55**)	*Trichoderma* sp. USF-2690 from a soil sample	[29]
Dihydrotrichodermolide (**56**)	*Phialocephala* sp. FL30r from a deep-sea sediment	[36]
Trichodermolide (**57**)	*Trichoderma longibrachiatum* UAMH 4159 and its origin was not clear	[26,54]
Tetrahydrotrichodimer ether (**58**)	*Clonostachys rosea* YRS-06 from a soil sample	[13]
Dihydrotrichodimer ether A (**59**)	*Clonostachys rosea* YRS-06 from a soil sample	[13]
Dihydrotrichodimer ether B (**60**)	*Clonostachys rosea* YRS-06 from a soil sample	[13]

Note: Compounds **36**, **39**–**41 and** **56**–**60** were not included in the last review [1].

**Table 3:** Occurrence of the trimeric sorbicillinoids (**61**–**65**) in fungi.

Sorbicillinoid	Fungus and Its Origin	Ref.
Trisorbicillinone A (**61**)	*Phialocephala* sp. FL30r from a deep-sea sediment	[55]
Trisorbicillinone B (**62**)	*Phialocephala* sp. FL31r from a deep-sea sediment	[56]
Trisorbicillinone C (**63**)	*Phialocephala* sp. FL32r from a deep-sea sediment	[56]
Trisorbicillinone D (**64**)	*Phialocephala* sp. FL33r from a deep-sea sediment	[56]
Sorbicillamine E (**65**)	*Penicillium* sp. F23-2 from a deep-sea sediment	[10]

Note: Compound **65** was not included in the last review [1].

**Table 4:** Occurrence of the hybrid sorbicillinoids (**66**–**90**) in fungi.

Sorbicillinoid	Fungus and Its Origin	Ref.
Rezishanone A (**66**)	*Penicillium notatum* from a benchtop contamination	[5]
Rezishanone B (**67**)	*Penicillium notatum* from a benchtop contamination	[5]
Rezishanone C = Sorbivinetone (**68**)	*Penicillium chrysogenum* isolated from the sponge *Ircinia fasciculata*	[21]
	*Penicillium notatum* from a benchtop contamination	[5]
	*Trichoderma viride* from the sponge *Agelas dispar*	[3]
	*Trichoderma* sp. isolated from the sponge *Agelas dispar*	[37]
	Unidentified fungus B00853 from a soil sample	[50]
Rezishanone D (**69**)	*Penicillium notatum* from a benchtop contamination	[5]
	Unidentified fungus B00853 collected from a soil sample	[50]
Spirosorbicillinol A (**70**)	*Trichoderma* sp. USF-4860 from a soil sample	[60]
Spirosorbicillinol B (**71**)	*Trichoderma* sp. USF-4860 from a soil sample	[60]
Spirosorbicillinol C (**72**)	*Trichoderma* sp. USF-4860 from a soil sample	[60]
Unnamed sorbicillinoid urea (**73**)	*Paecilomyces marquandii* BAFC 486 from a marine sediment	[57]
Chloctanspirone A (**74**)	*Penicillium terrestre* from a marine sediment	[58]
Chloctanspirone B (**75**)	*Penicillium terrestre* from a marine sediment	[58]
Sorbicatechol A (**76**)	*Penicillium chrysogenum* PJX-17 from a marine sediment	[59]
Sorbicatechol B (**77**)	*Penicillium chrysogenum* PJX-17 from a marine sediment	[59]
Sorbicillamine A (**78**)	*Penicillium* sp. F23-2 from a deep-sea sediment	[10]
Sorbiterrin A (**79**)	*Penicillium terrestre* from a marine sediment	[61]
JBIR-59 (**80**)	*Penicillium citrinum* SpI080624G1f01 from the sponge *Demospongiae* sp.	[43]
JBIR-124 (**81**)	*Penicillium citrinum* SpI080624G1f01 from the sponge Demospongiae sp.	[62]
Sorbifuranone A (**82**)	*Penicillium chrysogenum* E03-8/4 from the sponge *Tethya aurantium*	[35]
Sorbifuranone B (**83**)	*Penicillium chrysogenum* E03-8/4 from the sponge *Tethya aurantium*	[35]
Sorbifuranone C (**84**)	*Penicillium chrysogenum* E03-8/4 from the sponge *Tethya aurantium*	[35]
Sorbicillactone A (**85**)	*Penicillium chrysogenum* E01-10/3 from the sponge *Ircinia fasciculata*	[21]
	*Penicillium chrysogenum* R03-8/4 from the sponge *Tethya aurantium*	[35]
Sorbicillactone B (**86**)	*Penicillium chrysogenum* E01-10/3 from the sponge *Ircinia fasciculata*	[21]
Trichodermanone A (**87**)	*Trichoderma viride* from the sponge *Agelas dispar*	[3]
	*Trichoderma* sp. from the sponge *Agelas dispar*	[37]
Trichodermanone B (**88**)	*Trichoderma viride* from the sponge *Agelas dispar*	[3]
	*Trichoderma* sp. from the sponge *Agelas dispar*	[37]
Trichodermanone C (**89**)	*Trichoderma viride* from the sponge *Agelas dispar*	[3]
	*Trichoderma* sp. from the sponge *Agelas dispar*	[37]
Trichodermanone D (**90**)	*Trichoderma viride* from the sponge *Agelas dispar*	[3]
	*Trichoderma* sp. from the sponge *Agelas dispar*	[37]

Note: Compounds **74**–**79** were not included in the last review [1].

**Table 5:** Cytotoxic activity of the screened sorbicillinoids from fungi.

Sorbicillinoid	Cytotoxic Activity	Ref.
Sorbicllin (**5**)	IC_50_ of 12.7 μM on HL-60 (Leukemia) cell line.	[27]
	IC_50_s of 1.6 and 27.2 μM on HeLa and HepG2 cells, respectively.	[7]
	IC_50_s of 6.55 to 28.55 μM on HL-60, U937 and T47D cell lines.	[30]
6-Demethylsorbicillin (**7**)	IC_50_ of 23.9 μM on HL-60 cell line.	[27]
1-(2′-Hydroxy-4′-methoxy-5′-methylphenyl)-*E,E*-2,4-hexadien-1-one (**9**)	IC_50_s of 65.2 and 15.1 µM on MDA-MB-435 and SW-620 cell lines at 72 h, respectively.	[12]
5'-Formyl-2′-hydroxyl-4′-methoxy-(*E,E*)-sorbophenone (**10**)	IC_50_s of 1.5 and 0.5 µM on MDA-MB-435 (melanoma) and SW-620 (colon) cell lines at 72 h, respectively, IC_50_ of 3.1 µM on OSU-CLL (lymphocytic leukemia) cell line at 48 h.	[12]
Scalbucillin B (**12**)	IC_50_s of 67.9 and 16.0 µM on MDA-MB-435 and SW-620 cell lines at 72 h, respectively.	[12]
2′,3′-Dihydrosorbicillin (**13**)	IC_50_s of 7.4 and 44.4 μM on HeLa and HepG2 cells, respectively.	[7]
	IC_50_s of 9.19 to 21.93 μg/mL on various human cancer cell lines.	[4]
Dihydrodemethylsorbicillin (**17**)	IC_50_s of 0.1 and 4.8 μM on P388 and K562 cell lines, respectively.	[36]
5′-Formyl-2′-hydroxy-4′-methoxy-(*E*)-4-hexenophenone (**18**)	IC_50_s of 2.3 and 2.5 µM on MDA-MB-435 and SW-620 cell lines at 72 h, respectively.	[12]
(2*S*)-2,3-Dihydro-7-hydroxy-6,8-dimethyl-2-[(*E*)-prop-1-enyl]-chroman-4-one (**21**)	IC_50_ of 9.51 µg/mL on human breast cancer cell line MCF-7.	[4]
(2*S*)-2,3-Dihydro-7-hydroxy-6-methyl-2-[(*E*)-prop-1-enyl]-chroman-4-one (**22**)	IC_50_ of 7.82 µg/mL on human breast cancer cell line MCF-7.	[4]
(*E*)-6-(2,4-Dihydroxyl-5-methylphenyl)-6-oxo-2-hexenoic acid (**23**)	IC_50_s of 44.5 μM and 72.8 μM on HL-60 and P388 cell lines, respectively.	[6]
6-(2,4-Dihydroxyl-5-methylphenyl)-6-oxohexanoic acid (**24**)	IC_50_s of 81.2 μM and 52.5 μM on HL-60 and P388 cell lines, respectively.	[6]
2-(2′,3′-Dihydrosorbyl)-3,6-dimethyl-5-hydroxy-1,4-benzoquinone (**25**)	IC_50_s of 15.7 μM and 5.3 μM on P388 and A549 cell lines, respectively.	[19]
Bisvertinolone (**34**)	IC_50_ of 5.3 μM on HL-60 cell line.	[27]
16,,17-Dihydrobisvertinolone (**35**)	IC_50_s of 1.7 μM and 0.52 μM on P388 and A549 cell lines, respectively.	[19]
10,11-Dihydrobisvertinolone (**36**)	IC_50_ of 49 μM on HL-60 cell line.	[27]
Tetrahydrobisvertinolone (**37**)	IC_50_s of 16.7 μM on A549 cell line.	[19]
Trichodimerol = MS-182123 (**42**)	IC_50_ of 7.8 μM on HL-60 cell line.	[27]
	IC_50_s of 0.33 and 4.7 μM on P388 and A549 cell lines, respectively.	[47]
	IC_50_s of 6.55 to 28.55 μM on HL-60, U937 and T47D cell lines.	[30]
Dihydrotrichodimerol (**44**)	IC_50_ of 36.4 μM on HL-60 cell line.	[27]
	IC_50_s of 2.8 and 2.1 μM on P388 and A549 cell lines, respectively.	[47]
	IC_50_s of 3-34 μM on U373, A549, SKMEL-28, OE21, Hs683, and B16F10 cell lines.	[51]
Tetrahydrotrichodimerol (**45**)	IC_50_s of 8.8 and 4.3 μM on P388 and A549 cell lines, respectively.	[47]
Bislongiquinolide =Bisorbibutenolide = Trichotetronine (**49**)	IC_50_s of 4-22 μM on U373, A549, SKMEL-28, OE21, Hs683, and B16F10 cell lines.	[51]
Oxosorbiquinol (**53**)	IC_50_s of 8.9, 29.9, 103.5, 12.7 and 56.3 μM on HL-60, P388, A549, BEL7402 and K562 cell lines, respectively.	[2]
Dihydrooxosorbiquinol (**54**)	IC_50_s of 10.5, 40.3, 97.6, 31.8 and 68.2 μM on HL-60, P388, A549, BEL7402 and K562 cell lines, respectively.	[2]
Dihydrotrichodermolide (**56**)	IC_50_s of 11.5 and 22.9 μM on P388 and K562 cell lines, respevtively.	[36]
Trisorbicillinone A (**61**)	IC_50_s of 3.14, 9.10, 60.28 and 30.21 μM on HL-60, P388, BEL7402 and K562 cell lines, respectively.	[55]
Trisorbicillinone B (**62**)	IC_50_s of 77.1 and 88.2 μM on P388 and K562 cell lines, respectively.	[56]
Trisorbicillinone C (**63**)	IC_50_s of 78.3 and 54.3 μM on P388 and K562 cell lines, respectively.	[56]
Trisorbicillinone D (**64**)	IC_50_s of 65.7 and 51.2 μM on P388 and K562 cell lines, respectively.	[56]
Chloctansprirone A (**74**)	IC_50_s of 9.2 and 39.7 μM on HL-60 and A549 cell lines, respectively	[58]
Chloctansprirone B (**75**)	IC_50_ of 37.8 μM on HL-60 cell line.	[58]
Sorbicillactone A (**85**)	IC_50_ of 2.2 µg/mL on L5178y (murine leukemic lymphoblasts) cell line.	[21]

Note: “IC_50_” means the median inhibitory concentration.

**Table 6:** Antimicrobial activity of the screened sorbicillinoids from fungi.

Sorbicillinoid	Antimicrobial activity	Ref.
Oxosorbicillinol (**3**)	Weak antibacterial activity on *Staphylococcus aureus* and *Bacillus subtilis*.	[5]
Sohirnone B (**8**)	Weak antibacterial activity on *Staphylococcus aureus* and *Bacillus subtilis*.	[5]
5′-Formyl-2′-hydroxyl-4′-methoxy-(*E*,*E*)-sorbophenone (**10**)	Showed potent activity against *Aspergillus flavus* (NRRL 6541) and moderate activity against *Fusarium verticillioides* (NRRL 25457).	[33]
Scalbucillin B (**12**)	MIC value of 0.60 μg/mL (2.42 μM) against *Aspergillus niger.*	[12]
2′,3′-Dihydrosorbicillinol (**13**)	Weak antibacterial activity on *Staphylococcus aureus* and *Bacillus subtilis*.	[5]
Sohirnone A (**14**)	Weak antibacterial activity on *Staphylococcus aureus* and *Bacillus subtilis.*	[5]
1-(2′-Hydroxy-4′-methoxy-5′-hydroxymethylphenyl)-*E*-4-hexen-1-one (**16**)	Showed potent activity against *Aspergillus flavus* (NRRL 6541) and weak activity against *Fusarium verticillioides* (NRRL 25457).	[33]
5′-Formyl-2′-hydroxy-4′-methoxy-(*E*)-4-hexenophenone (**18**)	Strong antifungal activity on *Aspergillus niger* with MIC values of 0.04 μg/mL (0.16 μM).	[12]
Sorrentanone [=3-hydroxy-2,5-dimethyl-6-(1′-oxo-2′,4′-dienylhexyl)-1,4-benzoquione, **26**]	MIC values of 32, 16, 128, 32, 32 and 64 µg/mL on *Staphylococcus pneumoniae* A9585, *S. pyogenes* A9604, *Enterococcus faecalis* A20688, *S. aureus*/Hetero MR A27218, *S. epidermidis* A24548, and *S. haemolytic* A21638, respectively.	[18]
Dihydrotrichodimerol (**44**)	Strong antibacterial activity on *Bacillus megaterium* with MIC value of 25 μg/mL.	[13]
Tetrahydrotrichodimerol (**45**)	Strong antibacterial activity on *Bacillus megaterium* with MIC value of 12.5 μg/mL.	[13]
Bisvertinoquinol (**47**)	Weak antibacterial activity on *Staphylococcus aureus* and *Bacillus subtilis*.	[5]
Bisorbicillinol (**48**)	Weak antibacterial activity on *Staphylococcus aureus* and *Bacillus subtilis*.	[5]
Dihydrotrichodimer ether A (**59**)	Strong antibacterial activity on *Escherichia coli* with MIC value of 25 μg/mL.	[13]
Dihydrotrichodimer ether B (**60**)	Strong antibacterial activity on *Escherichia coli* and *Ballus subtilis* with MIC values of 50 μg/mL.	[13]
Rezishanones A (**66**)	Weak antibacterial activity on *Staphylococcus aureus* and *Bacillus subtilis*.	[5]
Rezishanone B (**67**)	Weak antibacterial activity on *Staphylococcus aureus* and *Bacillus subtilis*.	[5]
Rezishanone C = Sorbivinetone (**68**)	Weak antibacterial activity on *Staphylococcus aureus* and *Bacillus subtilis*.	[5]
Rezishanone D (**69**)	Weak antibacterial activity on *Staphylococcus aureus* and *Bacillus subtilis*.	[5]
	Strong antifungal activity on *Aspergillus niger* with MIC value of 0.05 μg/mL (0.20 μM)	[12]

Note: “MIC” means the minimum inhibitory concentration.

**Table 7:** Other biological activities of the screened sorbicillinoids from fungi.

Sorbicillinoid	Biological Activity	Ref.
6′-Hydroxyoxosorbicillinol (**4**)	Inhibitory activity on soybean lipoxygenase; Prostaglandin D2 and leucotriene B4 release suppression activity.	[22]
Bisvertinolone (**34**)	Inhibitory effect on β-l,6-glucan biosynthesis	[42]
Isobisvertinol (**38**)	Inhibitory effect on lipid droplet accumulation in mouse macrophages	[40]
Trichodimerol (**42**)	Inhibitory effect on bacterial endotoxin-induced production of tumor necrosis factor (TNF-α) in murine macrophages and human peripheral blood monocytes	[46]
	Inhibitory effect on lipopolysaccharide-induced eicosanoid secretion in THP-1 human monocytic cells	[66]
	Suppression of the production of tumor necrosis factor-α and nitric oxide in LPS-stimulate RAW264.7 cells	[50]
Dihydrotrichodimerol (**44**)	Activation of peroxisome proliferator-activated recptor γ (PPAR γ) with an ED_50_ of 80 ng/mL	[50]
	Suppression of the production of tumor necrosis factor-α and nitric oxide in LPS-stimulate RAW264.7 cells	[50]
	Effect on feeding perference of the aphid	[48]
Bislongiquinolide (**49**)	Effect on feeding perference of the aphid	[48]
Sorbiterrin A (**79**)	Inhibitory effect on acetylcholinesterase activity with IC_50_ value of 25 μg/mL	[61]

Note: “ED_50_” means the median effective dose. “IC_50_” means the median inhibitory concentration.
